# Ultrasonographic and clinicopathological features of pelvic yolk sac tumors in women: a single-center retrospective analysis

**DOI:** 10.3389/fonc.2024.1417761

**Published:** 2024-06-20

**Authors:** Mei Chen, Shengmin Zhang, Xiupeng Jia, Youfeng Xu, Yaping Wei, Shusheng Liao

**Affiliations:** ^1^ Department of Ultrasonography, The First Affiliated Hospital of Ningbo University, Ningbo, Zhejiang, China; ^2^ Department of Histopathology, Ningbo Clinical Pathology Diagnosis Center, Ningbo, Zhejiang, China; ^3^ Department of Ultrasonography, Ningbo Women and Children’s Hospital, Ningbo, Zhejiang, China; ^4^ Department of Ultrasound, The First Affiliated Hospital of Wenzhou Medical University, Wenzhou, Zhejiang, China

**Keywords:** ovarian, yolk sac tumor, ultrasound, CEUS, malignant germ cell tumor

## Abstract

**Objectives:**

Yolk sac tumors (YSTs) are rare and highly malignant ovarian malignancies that have a very poor prognosis. The aim of this study is to delineate the ultrasound and clinicopathological features of female pelvic YSTs to better understand the disease.

**Methods:**

This study was a retrospective analysis of the clinicopathological and ultrasound imaging data from 16 YST patients who received treatment at our hospital between January 2012 and August 2023. Then, the ultrasound imaging characteristics were compared with pathological findings.

**Results:**

Among the 16 patients, various degrees of serum AFP increase were observed, and CA125 levels increased in 58.33% (7 out of 12) of patients. Thirteen patients (81.25%) had tumors located in ovary, two patients (12.5%) had tumors located in the sacrococcygeal region, and one patient (6.25%) had tumors located in the mesentery. Pathologically, nine patients presented with simple yolk sac tumors and seven with mixed germ cell tumors. According to the ultrasound manifestations, YST lesions can be classified into three types. (1) the cystic type, was diagnosed in two patients who presented with a large cystic mass with regular morphology and clear boundary and dense liquid within the cyst; and (2) the cystic-solid mixed type, was diagnosed in 4 patients. On 2D ultrasound, the lesions showed a cystic-solid mixed echo, and color Doppler showed a rich blood flow signal in the solid region and cystic separation. made up of four cases. (3) In ten patients with the solid type, 2D ultrasound showed solid uniform echoes with clear boundaries. The “fissure sign” was observed in the lesion. Color Doppler displayed rich blood flow in the solid part, and PW showed low to moderate resistance index of artery (RI:0.21–0.63). On contrast-enhanced ultrasound (CEUS), rapid and high enhancement in the solid part and cystic separation was observed in 2 patients.

**Conclusions:**

Combining ultrasound features with clinical information and tumor markers provides reliable clues for the diagnosis of YST. The application of two-dimensional ultrasound and CEUS combined with patient tumor marker levels can provide a robust reference for determining the necessity of fertility-preserving surgery and postoperative chemotherapy, which can improve clinical decision-making and patient consultation.

## Introduction

Primary ovarian tumors are usually classified as epithelial, intercord or germ cell tumors, with germ cell tumors accounting for approximately 15–20% of all ovarian tumors (1). Germ cell tumors are further divided into benign and malignant types, with mature teratoma being the most common benign type and malignant germ cell tumors being the most common undifferentiated carcinoma types. Contrary to epithelial ovarian cancers, germ cell tumors mostly present at an earlier age in women, grow faster and present unilaterally (2). Yolk sac tumor (YST), also known as endodermal sinus tumor, is a rare and highly malignant germ cell tumor that accounts for 1% of all ovarian malignancies (3). Ovarian YSTs predominantly affect children and young women, with onset typically occurring before the age of 30; even some patients are pregnant or postmenopausal period (4, 5). It exhibits rapid growth, invasiveness, and a propensity for metastasis, resulting in a very poor prognosis. Before the 1970s, the three-year survival rate for patients was only 13% (6). However, advancements in diagnostic techniques, surgical methods, and adjuvant therapy, particularly the use of modern treatment regimens such as BEP (bleomycin, etoposide, cisplatin) have significantly improved the 5-year survival rate for patients with YSTs (7, 8). As life expectancy increases, patients, especially younger patients, have heightened expectations for quality of life, including concerns about fertility preservation after surgery. Therefore, early diagnosis through imaging methods is crucial for patients to achieve favorable outcomes. Ultrasonographic diagnosis plays an important role in imaging diagnosis, with contrast-enhanced ultrasound(CEUS), a new technique reflecting tumor microcirculation, holding particular significance in the early diagnosis of tumors (9–11). Due to the low incidence of YSTs, previous reports in the literature mainly consist of individual cases (12). Moreover, ultrasound reports have been limited to two-dimensional gray scale ultrasound (13), and there is a lack of literature on classifying YST basing on ultrasound images. In this study, a retrospective analysis of multimodality ultrasound manifestations of 16 patients with YSTs, combined with clinical data, was conducted to improve the diagnostic accuracy of YSTs.

## Materials and methods

### Study population

This retrospective study was conducted at Ningbo First Hospital and it adhered to the principles outlined in the Declaration of Helsinki. The study was approved by the ethics committee of Ningbo First Hospital (No.2023RS128). Individual consent was obtained from all patients in this retrospective analysis. A computerized search of the pathology records at the hospital was conducted from January 2012 to August 2023. This resulted in the identification of 27 patients with a histological diagnosis of YSTs. Subsequently, a second search was performed on the hospital’s imaging archiving and communication system to identify female patients who underwent 2D and/or CEUS examination prior to surgery. Ultimately, 16 patients were identified, 9 of whom were male. All 16 patients had 2D ultrasound examination data, and two of them also had CEUS results.

### Ultrasound examination

The equipment and parameters for this study included the GE E8, Toshiba Aplio 500, and Philips IU22 color Doppler ultrasound diagnostic systems. Ultrasound examinations were conducted using a transvaginal probe with a frequency range of 5–9 MHz and a transabdominal probe with a frequency range of 3.5–5.0 MHz. All patients underwent standard two-dimensional and color Doppler ultrasound assessments. The two-dimensional ultrasonography was used to evaluate the lesion location, size, shape, boundaries, internal echo characteristics, and the presence of pleuritic and abdominal effusion. Color Doppler flow imaging (CDFI) was utilized to analyze the blood flow within the lesions.

Additionally, two patients underwent contrast-enhanced ultrasound (CEUS) to investigate the microcirculation patterns within the lesions. This procedure involved the administration of a contrast agent (SonoVue, Bracco) at a 1.8 ml dose via the median antecubital vein. Throughout the contrast-enhanced phase, continuous observation was made of the enhancement pattern, timing, intensity, and washout of the contrast agent within the lesions.

### Statistical methods

Continuous data are presented as mean or median (interquartile spacing (IQR)), and categorical data are presented as frequencies and percentages. The analysis was performed using IBM SPSS 17.0 (IBM, Armonk, NY)

## Results

### Clinical data of the 16 patients

**Figure 1:**
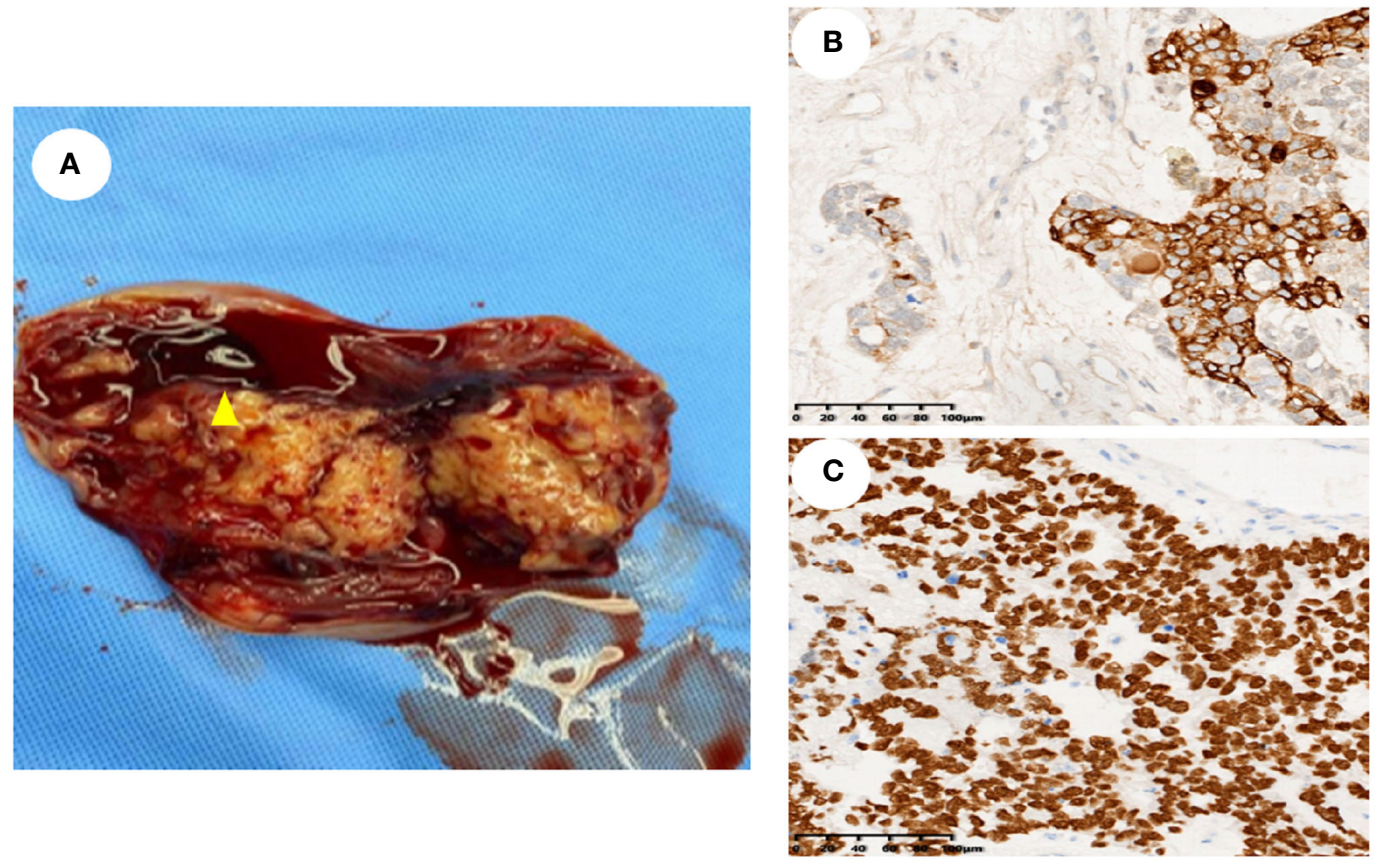
General sample and immunohistochemical markers of ovarian yolk sac tumor. **(A)**. A gross specimen of an ovarian yolk sac tumor was an oval mass with a yellow vision, a smooth capsule and visible capsular hemorrhage (triangle); **(B)**. Immunohistochemistry showed high AFP expression in yolk sac tumor cells (SP method, ×200); **(C)**. Immunohistochemistry showed high SALL4 expression in yolk sac tumor cells (SP method, ×200).

Sixteen female patients with a pathological diagnosis of YST were enrolled in this study. The clinical characteristics are shown in **Table 1**. The mean age of the 16 patients was 23.25 ± 20.80 (range: 1 ~72) years, 68.75% were younger than 30 years, while 31.25% were older than 30 years. Additionally, 12.5% (2 out of 16) were menopausal, and 87.5% (14 out of 16) were non-menopausal. The patients were follow-up for a mean duration of 22.5 ± 14.56 months (range: 3–54).Fourteen patients presented with abdominal pain, while two patients were asymptomatic. Serum AFP levels were increased in all (100%, 16 out of 16) patients, with 70% (7 out of 10) of patients showing higher CA 125 levels, 16.66% (2 out of12)of patients showing higher CEA levels, 36.36% (4 out of 11) of patients showing increased CA199 levels, and only 12.5% (1 out of 8) of patients showing higher CA724 levels. The CA153 level in 8 patients, along with the β-hCG level in 11 patients, was within the normal range. Employing the FIGO tumors staging system, this study revealed that 87.5% (14 out of 16) of patients were classified as stage I, 6.25% (1 out of 16) as stage III, and 6.25% (1 out of 16) as stage IV. Thirteen patients (81.25%) had tumors located in the ovary, two patients (12.5%) had tumors located in the sacrococcygeal region, and one patient (6.25%) had tumors located in the mesentery. Among these 16 patients, ten underwent transabdominal ovarian and adnexectomy, three received total and double appendicectomy, and three had only lesion resected. Gross specimen examination revealed a round or oval mass with a yellow color, clear capsule, and visible “cobblestone”, similar to irregular liquid dark areas. Some dark areas were brown (**Figure 1A**).

**Table 1:** Clinical characteristics of 16 patients with ovarian yolk sac tumor.

Parameters		Yolk Sac tumor (n = 16)
Age at surgery [years]		23 (1–72)
0–9		18.75% (3/16)
10–19		31.25% (5/16)
20–30		18.75% (3/16)
>30		31.25% (5/16)
Serum CA125 level [U/mL] Reference range(0–30)	<30	41.66% (5/12)
	>30	58.33% (7/12)
Serum CA-724 level [U/ml] Reference range(0–7)	<7	87.50% (7/8)
	>7	12.50% (1/8)
Serum CA-153 level [U/mL] Reference range(0–25)	<25	100% (8/8)
	>25	0
Serum CA-199 level [U/ml]Reference range(0–25)	<25	63.63% (7/11)
	>25	36.36% (4/11)
AFP level [ng/mL] Reference range(0–25)	<25	0
	>25	100% (16/16)
CEA level [U/mL] Reference range(0–10)	<10	83.33% (10/12)
	>10	16.66% (2/12)
Menstruation	Premenopausal	87.50% (14/16)
	Postmenopausal	12.50% (2/16)
Symptomatology	Asymptomatic	12.50% (2/16)
	Pelvic pain	87.50% (14/16)
Surgery	Transabdominal tylectomy	25.0% (4/16)
	Salpingo-oophorectomy laparotomy	56.25% (9/16)
	Total abdominal hysterectomy	18.75% (3/16)
FIGO by stages	I designated time	81.25% (13/16)
	II designated time	0
	III designated time	12.5% (2/16)
	VI designated time	6.25% (1/16)
pathology	Simplicity	56.25% (9/16)
	Mixed type	43.75% (7/16)

### Pathological characteristics of the patients

**Figure 2:**
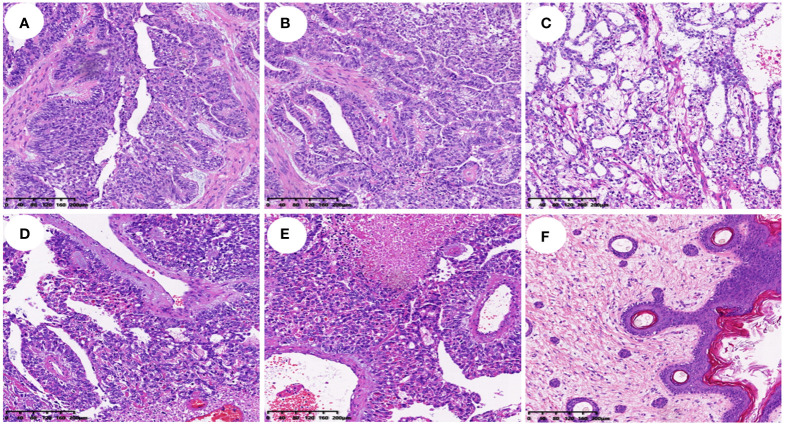
Histopathological findings of different types of yolk sac tumor (HE staining, all ×100). **(A–C)**. Histopathological findings of a simple yolk sac tumor; **(D–F)**. Histopathological findings of a mixed germ cell tumor with yolk sac tumor cell component in **(D)**, Schiller-Duval (SD) body in **(E)**, and mature teratoma in **(F)**.

Histopathological analysis revealed that nine (56.25%) patients had pure YSTs (**Figures 2A–C**), while seven (43.75%) patients had mixed germ cell tumors (**Figures 2D–F**). Among the mixed germ cell tumors, 25% (4 out of 16) had different combinations of YSTs and mature teratomas (**Figure 2F**); 12.5% (2 out of 16) had a combination of 10% YSTs and 90% immature teratomas, and one patient had poorly differentiated carcinoma with yolk sac differentiation. Among patients with serum AFP levels greater than 3000, 71.42% (5 out of 7) had pure yolk sac tumor, while 29.58% (2 out of 7) had a combination of 90% yolk sac tumors and 10% mature teratomas. Regarding immunohistochemical markers in the 16 patients, 93.75% (15 out of 16) of the patients were AFP positive (**Figure 1B**). One hundred percent (10 out of 10) of patients were CK (pan) positive, and 100% (13 out of 13) of patients were SALL 4 (+) positive (**Figure 1C**). One hundred percent (12 out of 12) of patients were positive for GPC 3, 91.66% (11out of 12) of patients were positive for HNF1β and 100% (7 out of 7) of patients were positive for Lin28. The Ki-67 (%) ranged between 25% and 90% in 15 patients. All 12 patients were tested negative for D2–40 and β-HCG, while 90.90% (10 out of 11) of the patients tested negative for NapsinA. The immunohistochemistry results are shown in **Table 2**.

**Table 2:** Two-dimensional and color Doppler ultrasound findings of the 16 patients with YSTs.

Ultrasonic classification		Cystic (n=2)	Cystic-solid Mixed (n=4)	Solid (n=10)
Number		12.50% (2/16)	25% (4/16)	62.50% (10/16)
Position	Left		12.50% (2/16)	31.25% (5/16)
	Right	12.50% (2/16)	12.50% (2/16)	12.50% (2/16)
	Other			18.75% (3/16)
Maximum diameter (cm)		9.6~12.1	10.7~25	6.9~16.9
Shape	Regular	12.50% (2/16)	18.75% (3/16)	38.45% (7/16)
	Irregular	0	6.25% (1/16)	43.75% (3/16)
Border	Clear	12.50% (2/16)	25% (4/16)	50% (8/16)
	Unclear			12.50% (2/16)
Cystic fluid	Clear	6.25% (1/16)		
	Unclear	6.25% (1/16)	25% (4/16)	12.50% (2/16)
	No			50.0% (8/16)
Separated	Yes	6.25% (1/16)	25% (4/16)	12.50% (2/16)
	No	6.25% (1/16)		50.0% (8/16)
Parenchymal echo and blood flow in the sac	Yes	6.25% (1/16)	25% (4/16)	0
	No	6.25% (1/16)	0	62.50% (10/16)
RI				0.21–0.63
Calcification	Yes		0	
	No	12.50% (2/16)	25.0% (4/16)	62.50% (10/16)
Associated with ascites	No		6.25% (1/16)	25.0% (4/16)
	Mild	12.50% (2/16)	12.5% (2/16)	12.5% (2/16)
	Medium			12.50% (2/16)
	Severe		6.25% (1/16)	12.50% (2/16)
Associated with hydrothorax	Yes	6.25% (1/16)		
	No	6.25% (1/16)	25% (4/16)	62.50% (10/16)
Associated with uterine fibroids	Yes	6.25% (1/16)		6.25% (1/16)
	No	6.25% (1/16)	25% (4/16)	56.25% (9/16)
Associated with Endometrial polyp	Yes	6.25% (1/16)		
	No	6.25% (1/16)	25% (4/16)	62.50% (10/16)
Ultrasound diagnosis	Benign	12.50% (2/16)	6.25% (1/16)	
	Uncertain			18.75% (3/16)
	Malignant		18.75% (3/16)	43.75% (7/16)

### Ultrasound classification and image characteristics of 16 patients

The ultrasound findings are shown in **Table 3**. All 16 patients presented with solitary tumors, including seven in the left ovary, six in the right ovary, two in the sacrococcygeal region and one in the mesentery. According to the ultrasound image of the tumor, it was divided into cystic, cystic-solid mixed type and solid type.

**Figure 3:**
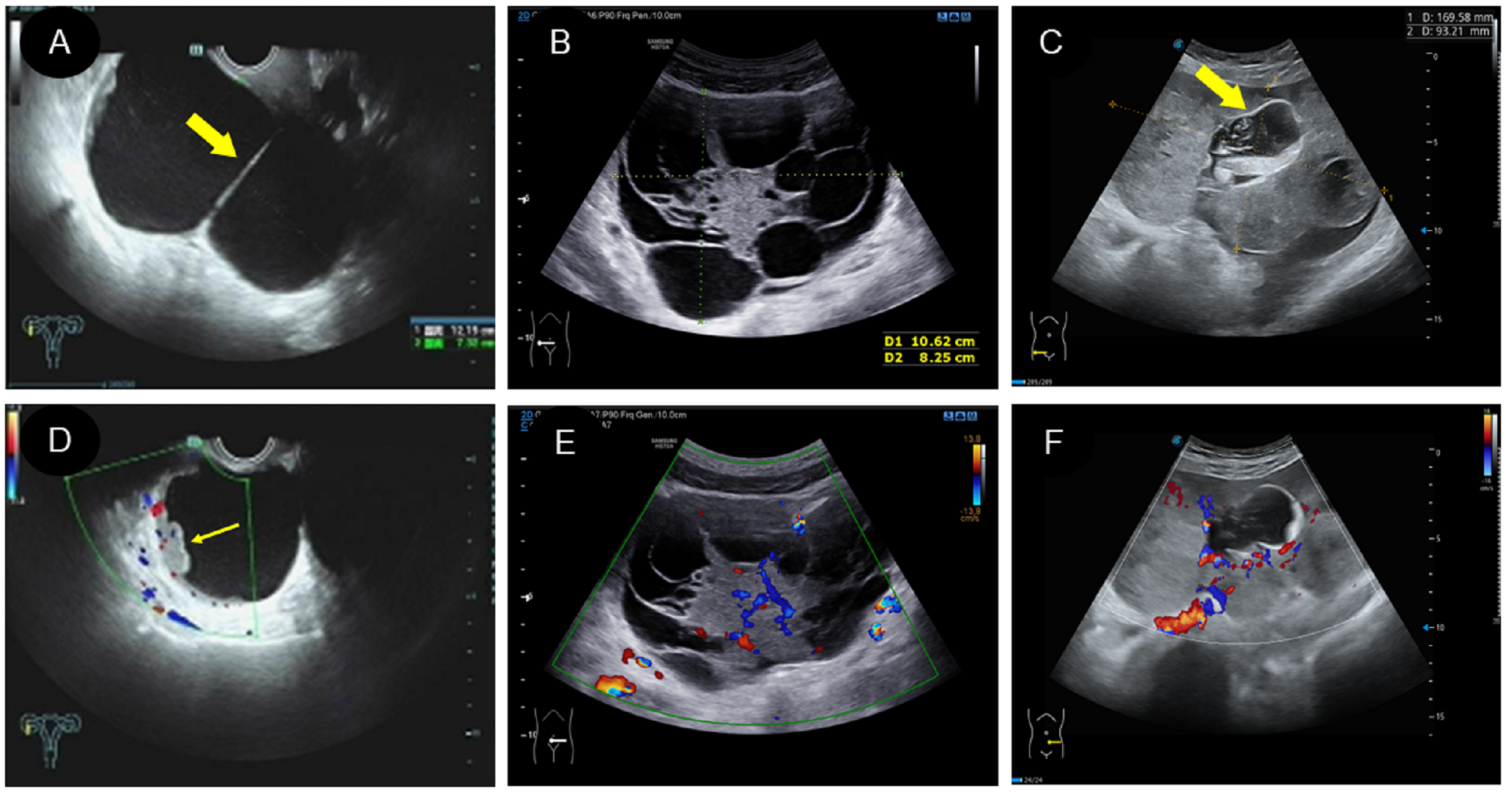
Two dimensional and color Doppler ultrasound findings of different types of yolk sac tumors. **(A, D)**. Cystic yolk sac tumor is a large cyst with regular morphology and clear boundary. The cyst is filled with weak echoes, with septation (thick arrow) and mural papillary nodules (thin arrow) visible on the wall. Color Doppler flow imaging (CDFI) showed punctate blood flow signals in the cystic wall and nodules; **(B, E)**. Cystic solid mixed yolk sac tumor also showed regular morphology and a clear boundary. The solid part showed low echoes, while the cystic part displayed large separation of different sizes and uneven thickness, separating the cystic area into many small chambers. CDFI revealed rich blood flow signals in the solid part and separation; **(C, F)**. Solid yolk sac tumor appears as a large solid mass with a more irregular morphology but a clear boundary. It displayed moderate echogenicity, less uniformity, and partially visible small cystic area (thick arrow), CDFI showed rich internal flow signals.

**Figure 4:**
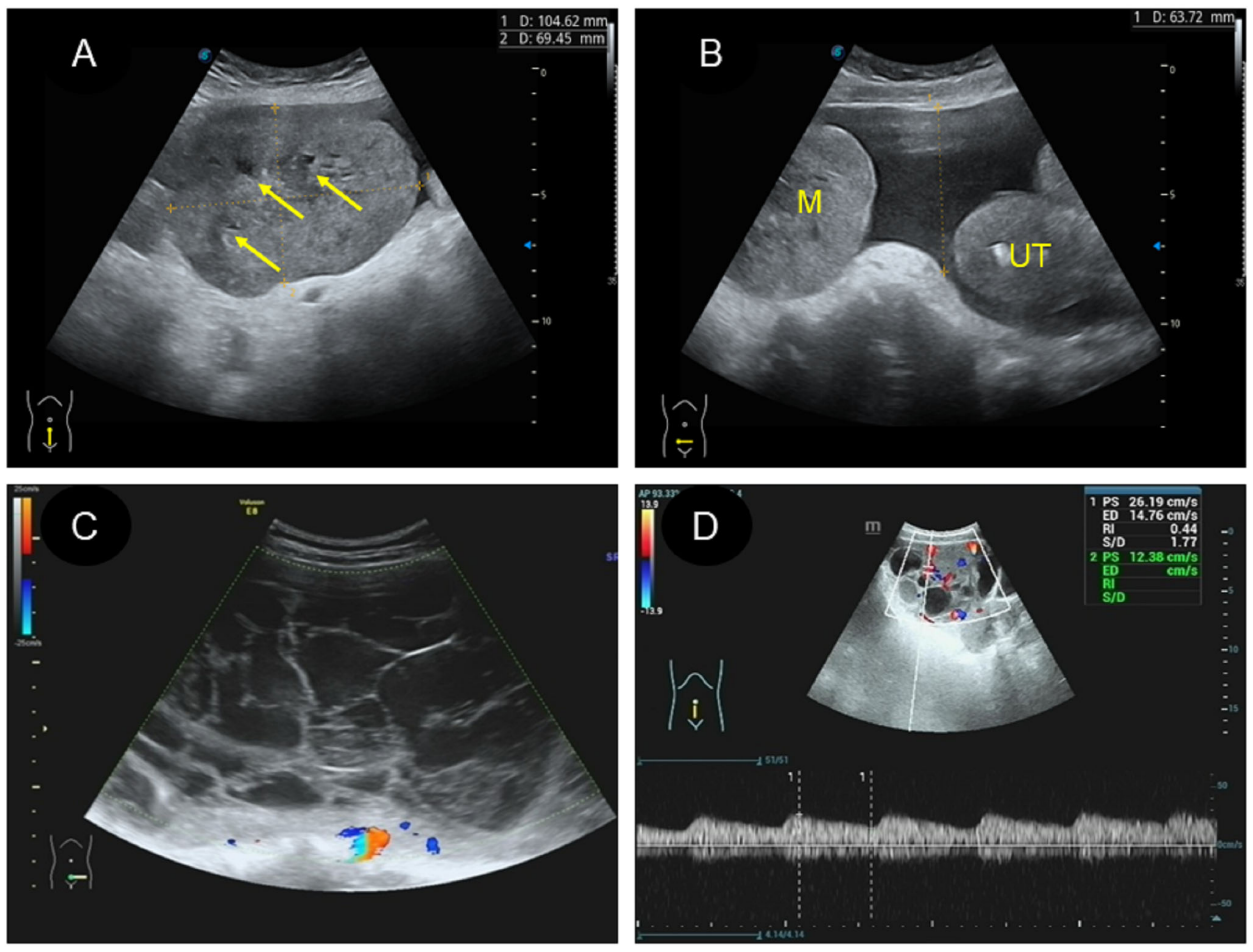
Some specific ultrasound findings of different types of yolk sac tumor. **(A)**.” Fissure sign “ inside the solid yolk sac tumor (thin arrow); **(B)**. A solid yolk sac tumor complicated with a large amount of ascites; **(C)**. A large number of irregular arrangement separation in cystic type of yolk sac tumor, and CDFI showed no blood flow signal in separation; **(D)**. PW showed low resistance of artery in the solid type of yolk sac tumor.

(1) Two patients had the cystic type (**Figure 3A**; **Figure 4C**), with a maximum diameter of 12.1cm, regular morphology and clear boundary. One patient presented with a cyst filled with dense liquid; the other patient presented with a cyst with septation and mural papillary protrusion, and color Doppler imaging revealed punctate blood flow signal in septation and papillary (**Figure 3D**). Two patients had moderate ascites, and one patient had pleural effusion.
(2) There were four cases of the cystic-solid mixed type (**Figure 3B**), with a maximum diameter of 25cm, clear boundary and regular morphology, and cystic part was filled with fine light spot echoes. Color Doppler demonstrated a rich blood flow signal in solid part and cystic separation (**Figure 3E**). One patient had a large amount of ascites, two patients had moderate ascites. One patient was classified as a benign tumor according to ultrasound performance, and three cases as malignant.
(3) There were 10 patients with the solid type (**Figure 3C**), with a maximum diameter of 16cm. Among them, three patients had irregular morphology, and two had unclear boundaries. The “fissure sign” was observed in lesions (**Figure 4A**). Color Doppler showed rich blood flow in the solid region (**Figure 3F**), and the PW showed a low to moderate resistance index of artery (RI:0.21–0.63) (**Figure 4D**). Six patients exhibited various degree of ascites (**Figure 4B**). One patient accompanied with uterine fibroids. No calcification was observed in any of the lesions.

**Table 3:** Expression of immune markers in tumor cells of 16 patients with yolk sac tumor.

Immunohistochemical markers	AFP	CK (pan)	SALL4(+)	GPC3	CK7	HNF1β	CD 30	CD 117	D2–40	HCG-β	Lin28	NapsinA	Ki-67 (%)
Case1	+	+	+	+			–	–	–	–	+		80
Case2	+	+	+	+	–	+	–	–	–	–	+	–	70
Case3	+	+			+	+						+	70
Case4	+		+	+	–	+	–	–	–	–		–	80
Case5	+	+	+	+	–	+	–	+	–	–		–	25
Case6	+	+		+	–	+		+	–	–		–	80
Case7	+		+	+	–	+	–	+	–	–	+	–	80
Case8	+	+++	+				–	+					80
Case9	+		+	+	–	+	+			–	+		80
Case10	+	+	+	+	–	+	–	+	–	–	+	–	60
Case11	–				+	–						–	70
Case12	+		+	+	–	+	–	+	–	–	+	–	90
Case13	+		+	+	–	+	–	+	–	–		–	40
Case14	+	+	+	+	+	+		+	–	–	+	–	60
Case15	+	+	+				–	+	–				
Case16	+	+	+	+			–	+	–	–			60

+, positive; +++, strongly positive; -, negative.

A and D. Cystic yolk sac tumor is a large cyst with regular morphology and clear boundary. The cyst is filled with weak echoes, with septation (thick arrow) and mural papillary nodules (thin arrow) visible on the wall. Color Doppler flow imaging (CDFI) showed punctate blood flow signals in the cystic wall and nodules; B and E. Cystic solid mixed yolk sac tumor also showed regular morphology and a clear boundary. The solid part showed low echoes, while the cystic part displayed large separation of different sizes and uneven thickness, separating the cystic area into many small chambers. CDFI revealed rich blood flow signals in the solid part and separation; C and F. Solid yolk sac tumor appears as a large solid mass with a more irregular morphology but a clear boundary. It displayed moderate echogenicity, less uniformity, and partially visible small cystic area (thick arrow), CDFI showed rich internal flow signals.

### CEUS image characteristics in two patients

**Figure 5:**
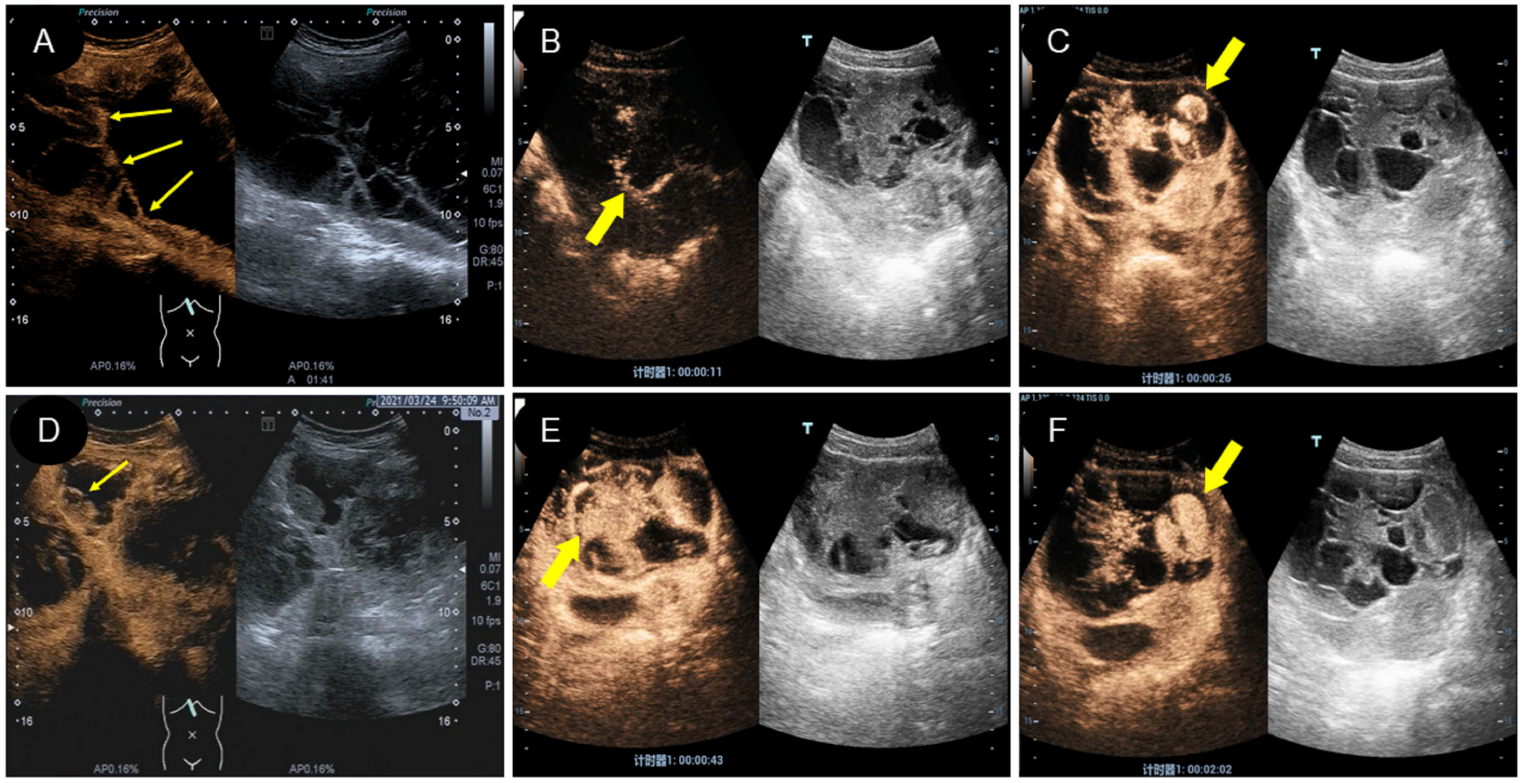
Characteristics of CEUS different types of yolk sac tumor. **(A, D)**. A mixed yolk sac tumor showed rapid contrast enhancement and no contrast perfusion in the sac (thin arrow); **(B, E)**. A solid yolk sac tumor showed rapid enhancement of solid and cystic area separation, with large internal vessels in the early stage (thick arrow) and uniform high enhancement after peak (thick arrow); **(C, F)**. In this case of a solid yolk sac tumor, the contrast agent flooded into and accumulated into the sac wall (thick arrow) after a burst, leaving a large number of contrast agent remained in the cystic cavity (thick arrow) after the resolution of the solid part.

Two of the 16 patients in this study underwent CEUS. Patient 1 was of cystic-solid mixed type, and after contrast agent injection, the solid and separated parts were rapidly enhanced and quickly subsided (**Figures 5A, D**; **Supplementary Video 1**). There was no contrast agent entry into the capsule, and no contrast echo was observed in the pelvic fluid. Patient 2 had a 17-year-old solid lesion with rapid contrast enhancement after contrast agent injection (**Figures 5B, E**; **Supplementary Video 2**). One of the cystic separations showed contrast accumulation, and the contrast agent flooded into and accumulated into the sac wall after a burst, leaving a large amount of contrast agent in the cystic cavity (**Figures 5C, F**). However, there was no contrast agent in the pelvic cavity. The patient stood up after ultrasound examination and then experienced shock. There was an intraoperative opening in the tumor, and a large amount of bloody fluid were observed in the pelvic cavity.

## Discussion

Pathologically, our study revealed that yolk sac tumors can present as pure or mixed germ cell tumors. A significantly higher AFP level was the diagnostic biomarker for YSTs. YST can present as cystic, cystic-solid or solid profiles on two-dimension ultrasound, and each type has distinct features. Rich blood flow in the solid part at color Doppler examination is another important ultrasound feature. Exploratively, rapid and high enhancement in the solid part was observed in two YST patients by using CEUS. Therefore, an important strength of this study is that the integration of conventional ultrasound and CEUS methods was introduced to elaborate the characteristics of YSTs. A second strength is that the combination of ultrasound features and biomarkers has been emphasized in the diagnosis of YSTs.

In 1939, Schiller named the characteristic reticuloglomerular-like structure of yolk sac tumors and described the tumors as originating in the middle kidney (8, 14). In 1959, it was discovered that the endodermal sinus of rat placenta was derived from extraembryonic germ cells (15). Studies by Kojimahara (7) and Nasioudis (16) confirmed that yolk sac tumors typically present unilaterally, are large and present often in the early stages of the disease (17). In this study, 87.5% (14 out of 16) of patients were diagnosed with stage FIGO I tumors, with tumor diameters ranging from 6.5cm to 25.0cm. Interestingly, there have reports of successful pregnancies in patients with YSTs, with some patients continuing pregnancy and experiencing smooth deliveries after surgery, as reported by Alrjoub (18) and Pafilis (19). YSTs are more common in female ovaries or male testis, but they can also manifest along the migration path of testicular tissue or in unusual locations, such as the vulva, mediastinum, sacral tail, mesentery, and even intracranial sites, such as the nasopharynx, sinuses, and pineal body (20). Pathologically, YSTs can present as simple lesions or as a part of mixed germ cell tumors (17, 21). Malignant mixed germinoma are defined by the presence of two or more malignant germ cell components, including yolk sac tumors, embryonal carcinomas, choriocarcinomas or immature teratomas (7, 22). Among these, the combination of asexual cell tumor and yolk sac tumor is the most common (23). Therefore, pathology reports should elaborate on the sizes and percentages of all germ cell components (22).

In this study, 87.50% (14 out of 16) of patients presented with abdominal pain, and 12.50% (2 out of 16) had no obvious symptoms, which is consistent with the findings of the study by Faure et al. (17). This discrepancy in symptoms may be attributed to the size and aggressiveness of the tumor. Asymptomatic patients likely have smaller tumors in the early stages, whereas those experiencing abdominal pain and distension may have larger, rapidly growing tumors that compress surrounding organs. In addition, the aggressive nature of the tumor can lead to thoracic or abdominal metastasis, resulting in pleural fluid or ascites. Among the 16 patients in this study, 11 had various degrees of ascites. All 16 patients underwent surgical procedures, with 56.25% (9 out of 16) presenting with pure YST, and 43.75% (7 out of 16) with mixed germ cell tumors, which is consistent with previous reports (17). Within the mixed germ cell tumors subgroup, 25% (4 out of 16) had different combinations of YSTs and mature teratomas, while 12.5% (2 out of 16) had a 10% YST and 90% immature teratoma combination, along with one case of poorly differentiated carcinoma with yolk sac differentiation. These findings contrast with those of Ulbright (14), who reported that mixed germ cell tumors are common in patients with yolk sac tumor. Post-surgery tumor staging revealed that 81.25% (13 out of 16) of patients had stage FIGO I tumors, which were confined to one side of the ovary, suggesting an early stage despite a larger tumor size. After surgery, patients received BEP chemotherapy, and after a follow-up period of 22.5 ± 14.56 (ranging from 3 to 54 months), none of the patients experienced recurrence or metastasis. Comprehensive treatment has significantly improved the 5-year survival rate for female YST, across FIGO stages I, II, III and IV, and the percentages for each stage are 94.8%, 97.1%, 70.9% and 51.6% respectively (7). Even patients with advanced FIGO stage disease benefited from comprehensive treatment for fertility protection, underscoring its efficacy in prolonging life and improving the quality of life. Therefore, timely detection through imaging methods and laboratory examination forms the cornerstone for achieving favorable prognosis.

Due to their origin from primordial germ cells, nearly all YST patients exhibit positive expression of serum AFP, which is a fundamental diagnostic tool and prognostic indicator for the tumor (24). In this study, all 16 patients demonstrated elevated AFP levels, with 43.75% (7 out of 16) showing levels greater than 3000. Postoperative pathology confirmed that 71.42% (5 out of 7) of patients had pure yolk sac tumors, while 29.58% (2 out of 7) had predominantly yolk sac tumors with a minor component of mature teratoma. The level of AFP correlates closely with the tumor pathology; pure yolk sac tumors show significantly higher AFP levels than mixed germ cell tumors, aiding in preoperative tumor classification and postoperative efficacy and recurrence assessment. Although YST is mostly present at early age of women, but an ovarian mass with elevated serum AFP level in postmenopausal women should suspect the diagnosis of germ cell tumors (5). In this study, 70% (7 out of 10) of patients exhibited various degrees of CA125 elevation, potentially linked to enteroid cells with multiphase differentiation potential (25). However, the specificity of CA125 in yolk sac tumors is less strong. Recent literature has indicated that combination of various serum biomarkers and patient characteristics may provide effective screening modalities for ovarian cancer (26). Currently, CA-125 and HE4 are considered two important biomarkers of gynecological malignancies and are mostly found in epithelial-derived tumors (26). However, these methods are not sufficient for early detection of pelvic masses. Conversely, the serum levels of CEA, CA724, CA153, and β-hCG did not significantly increase in this study.

Regarding the immunohistochemical markers in this cohort, 100% (10 out of 10) were positive for CK (pan), 100% (13 out of 13) were positive for SALL 4 (+), 100% (12 out of 12) were positive for GPC 3 and 100% (7 out of 7) were positive for Lin28.This finding confirms the significance of these markers as strong and specific indicators for YSTs, which is consistent with the findings of previous literature (27). Additionally, 93.75% (15 out of 16) of patients tested positive for AFP, with the exception of Patient 11, who had a serum AFP level of 12.95ng/mL. This patient was identified as having undifferentiated cancer with a small amount of yolk sac differentiation, indicating that a negative AFP result on immunohistochemistry cannot completely exclude the possibility of yolk sac tumor components, possibly due to their limited presence in the tumor. SALL 4 (+) is a more representative immune marker than AFP (28), with all 13 patients in this study testing positive. There are also studies suggesting that HNF1β is a new indicator of YST (29). In this study, 91.66% (11out of12) of patients were tested positive for HNF1β. Furthermore, 80.0% (12 out of15) patients were tested positive for CD117, and in the case of Ki-67 (%), which ranged between 25% and 90%, a positive correlation was observed with the content of yolk sac tumor component, showing its potential as an important immunohistochemical indicator. Conversely, 100% (12 out of 12) tested negative for D2–40 and HCG- β, and 90.9% (10out of11) tested negative for NapsinA, providing a basis for the differential diagnosis of YST.

This study recorded the clinical data and two-dimensional ultrasound findings of 16 patients with YSTs, attempting to classify and elaborate the characteristics of each subtype, while also documenting the CEUS findings in two patients. The lesions were classified into three types according to ultrasound findings: (1) the cystic type, diagnosed in 2 patients, one of whom showed septation and nodules in the sac. This type is easily confused with cystadenoma of the ovary (13). Although it can appear as a large cyst with intracapsular septa and protrusion, the serum AFP of patients with cystadenoma of the ovary was negative. (2) The cystic-solid mixed type showed a well-defined mass with cystic separation of varying thickness, an irregular morphology of solid part, and rich blood flow, as observed on color Doppler imaging. This ultrasonographic manifestation may be attributed to rapid tumor growth and the imperfect development of capillaries, resulting in internal ischemic necrosis. In this study, one patient was erroneously classified as a benign tumor, while three patients were deemed malignant. It is challenging to identify YST only by ultrasound images, especially those with rich blood flow, and it is difficult to distinguish them from ovarian cystadenocarcinoma (30) and ovarian metastases. Ovarian cystadenocarcinoma usually occurs at an older age, and serum AFP is negative, whereas metastatic tumors have a history of primary malignancy in other organs.

(3) The solid type showed slightly more solid echoes, fine inner texture, and “crack” and “cheese” sign, with a low resistance index. Seven patients were considered possibly malignant, while three patients were difficult to diagnose. The “fissure sign” observed in ultrasound manifestations is attributed to rapid tumor growth, immature development of internal blood vessels, and the band-like ischemic necrosis in the weak areas with limited blood supply. The difficulty in determining the nature of these three tumors may stem from their smooth boundaries and lack of invasive breakthrough envelope growth characteristics. Ascites in these patients result from the large mass compressing surrounding tissues, obstructing lymphatic reflux (13). This type of tumor should be distinguished from various solid ovarian tumors, such as subserous fibroids, broad ligament myoma, granulosa cell tumor. During the ultrasound examination, the echo of the tumor was found in the adnexa area, and normal acoustic images of the ovary were visible. Moreover, CDFI showed that the color blood flow signal extended from the uterine wall to the interior of the tumor, and the lesion may be a uterine subserous myoma or broad ligament myoma. Granulosa cell tumors have elevated level of estrogen and testosterone or androgen (31). In summary, when differentiating YSTs from other benign and malignant ovarian tumors based on ultrasound images is difficult, the patient’s age of onset, clinical manifestations and serum AFP can be used as important references for improving diagnosis.

CEUS plays an important role in reflecting the microcirculation within the tumor (32). In this study, two patients underwent CEUS examination. One patient was a 24-year-old female with a solid mixture tumor, exhibiting rapid and high enhancement after the injection of contrast agent. However, there was no contrast agent perfusion in the cystic area. Although the ultrasound images were similar to those of ovarian serous or mucinous tumors (33), these tumors were negative for serum AFP. The other patient, a 17-year-old woman with a solid tumor displayed rapid enhancement of the solid part and separation after contrast agent injection, followed by rapid subsidence. During this process, the contrast agent accumulated in the cystic cavity, with the tumor capsule remaining intact and no contrast agent distributed in the pelvic cavity. However, the patient experienced syncope immediately after the CEUS procedure, which was caused by the rupture of the tumor’s anterior and posterior walls and the presence of a large amount of bloody fluid in the pelvic cavity, which was identified after surgery. Retrospective analysis of the ultrasound contrast images revealed no contrast in the pelvic fluid, indicating damage to the cyst wall with contrast influx, despite smooth and intact tumor capsule observed in some patients’ general specimen. Real-time contrast-enhanced ultrasound can identify the location and extent of blood vessel damage in the cystic wall (34). With increasing bleeding in the tumor, tension can stimulate the capsule and cause lower abdominal pain. Abrupt increases in abdominal pressure, such as changes in the patient’s position or external forces, can lead to rupture of the tumor. Evaluating the distribution of contrast agent helps to assess tumor torsion and local bleeding, guiding clinicians on optimal timing for surgery.

## Conclusion

Combining ultrasound features with clinical information and tumor markers provides reliable clues for the diagnosis of yolk sac tumor. CEUS aids clinicians in assessing internal microcirculation and detecting internal bleeding. The integration of two-dimensional ultrasound and CEUS findings with patient tumor marker levels strengthens the decision-making process regarding the necessity of fertility-preserving surgery and postoperative chemotherapy, enhancing clinical outcomes and patient counseling. Due to the limitation of sample size in this study, a multicenter, large sample study is needed to further increase the understanding of this topic.

## Data availability statement

The raw data supporting the conclusions of this article will be made available by the authors, without undue reservation.

## Ethics statement

The studies involving humans were approved by the ethics committee of Ningbo First Hospital (No.2023RS128). The studies were conducted in accordance with the local legislation and institutional requirements. Written informed consent for participation in this study was provided by the participants' legal guardians/next of kin. Written informed consent was obtained from the individual(s), and minor(s)' legal guardian/next of kin, for the publication of any potentially identifiable images or data included in this article.

## Author contributions

MC: Conceptualization, Formal analysis, Funding acquisition, Investigation, Methodology, Resources, Validation, Writing – original draft, Writing – review & editing. SZ: Data curation, Investigation, Methodology, Project administration, Resources, Visualization, Writing – review & editing. XJ: Conceptualization, Methodology, Project administration, Resources, Supervision, Validation, Writing – review & editing. YX: Conceptualization, Methodology, Project administration, Validation, Visualization, Writing – review & editing. YW: Data curation, Project administration, Resources, Visualization, Writing – review & editing. SL: Conceptualization, Investigation, Project administration, Supervision, Visualization, Writing – original draft, Writing – review & editing.
